# Periostin Is a Key Niche Component for Wound Metastasis of Melanoma

**DOI:** 10.1371/journal.pone.0129704

**Published:** 2015-06-17

**Authors:** Keitaro Fukuda, Eiji Sugihara, Shoichiro Ohta, Kenji Izuhara, Takeru Funakoshi, Masayuki Amagai, Hideyuki Saya

**Affiliations:** 1 Division of Gene Regulation, Institute for Advanced Medical Research, Keio University School of Medicine, Tokyo, Japan; 2 Department of Laboratory Medicine, Saga Medical School, Saga, Japan; 3 Division of Medical Biochemistry, Department of Biomolecular Sciences, Saga Medical School, Saga, Japan; 4 Department of Dermatology, Keio University School of Medicine, Tokyo, Japan; IDI, Istituto Dermopatico dell'Immacolata, ITALY

## Abstract

Tissue injury promotes metastasis of several human cancers, although factors associated with wound healing that attract circulating tumor cells have remained unknown. Here, we examined the primary and metastatic lesions that appeared 1 month after trauma in a patient with acral lentiginous melanoma. The levels of mRNA for periostin (*POSTN*), type 1 collagen, and fibronectin were significantly increased in the metastatic lesion relative to the primary lesion. The increase of these extracellular matrix proteins at the wound site was reproduced in a mouse model of wound healing, with the upregulation of *Postn* mRNA persisting the longest. POSTN was expressed in the region surrounding melanoma cell nests in metastatic lesions of both wounded mice and the patient. POSTN attenuated the cell adhesion and promoted the migration of melanoma cells without affecting their proliferation *in vitro*. In the mouse model, the wound site as well as subcutaneously injected osteoblasts that secrete large amounts of POSTN invited the metastasis of remotely-transplanted melanoma cells on the sites. Osteoblasts with suppression of POSTN by shRNA showed a greatly reduced ability to promote such metastasis. Our results suggest that POSTN is a key factor in promoting melanoma cell metastasis to wound sites by providing a premetastatic niche.

## Introduction

The development of metastatic lesions at sites of tissue injury has long been recognized for several types of cancer [1]. The observation that melanoma metastases often appear at wound sites within a few months of trauma suggests that the wound healing process might establish a favorable microenvironment for the adhesion, migration, or proliferation of circulating melanoma cells and thereby promote their colonization [2–4]. However, whether injury actually promotes melanoma metastasis has not been demonstrated experimentally, and the factors and mechanisms associated with the wound metastasis remain to be elucidated.

The extracellular matrix (ECM) proteins type I collagen (COL-I) and fibronectin (FN) form fibrils and contribute to granulation tissue during wound healing [5, 6]. POSTN interacts directly with COL-I, FN, and tenascin-C and organizes the extracellular meshwork, conferring structural integrity on tissues subjected to mechanical stress [7]. POSTN is a matricellular protein, a class of nonstructural ECM proteins that modulate cell–matrix interactions, and expressed in tissues that are subject to mechanical stress such as periosteum, periodontal ligament, and heart valves [8]. It has recently been reported to serve as a niche for various tissue-specific stem cells including skin, mammary glands, and intestine [8, 9]. In the skin, its expression is localized to the basement membrane under steady state conditions but is also induced in granulation tissue [10, 11]. Mice deficient in POSTN manifest a phenotype of delayed re-epithelialization, revealing an important role for this protein in wound healing [12]. Intricate fibrillar networks composed of POSTN, COL-I, and FN are also assembled in lymph node macrometastases of melanoma, whereas such networks are rarely observed in lymph node micrometastases [13], suggesting that the network formation is associated with the aggressiveness of metastasis.

We now show that POSTN, COL-I, and FN components of fibrillar networks were upregulated in a metastatic lesion that developed at a wound site in a patient with melanoma. On the basis of this clinical experience, we examined which ECM proteins produced during wound healing may contribute to the induction of wound metastasis in melanoma and identified POSTN as a key factor attracting melanoma cells to injured sites.

## Materials and Methods

### Cell lines

The murine melanoma cell line B16-BL6, B16, and murine osteoblastic cell line MC3T3-E1 were obtained from RIKEN Cell Bank (Tsukuba, Japan). B16-BL6 cells were cultured in RPMI 1640 medium (Invitrogen, Carlsbad, CA) supplemented with 10% FBS. B16 cells were cultured in DMEM (Invitrogen) supplemented with 10% FBS. MC3T3-E1 cells were cultured in MEMα (Invitrogen) supplemented with 10% FBS. The human melanoma cell line MeWo and human breast cancer cell line Hs578T were obtained from American Type Culture Collection (Manassas, VA). MeWo cells were cultured in MEM (Invitrogen) supplemented with 2 mM L-glutamine (Invitrogen), 1% nonessential amino acids (Invitrogen), 1 mM sodium pyruvate (Invitrogen), and 10% FBS. Hs578T cells were cultured in DMEM supplemented with 10% FBS. The human melanoma cell lines WM35 and WM3734 were obtained from Coriell Institute for Medical Research (Camden, NJ) and were cultured in Tu2% medium, which consists of MCDB 153 medium (Sigma, St Louis, MO), L15 medium (Sigma), 2% FBS, 5 μg/ml insulin (Sigma) and 1.68 mM calcium chloride [14]. All cells were maintained at 37°C in a humidified atmosphere of 5% CO_2_.

### Preparation of conditioned medium

MC3T3-E1 cells were plated at 25% confluence in a 10-cm dish and cultured for 72 h in 8 ml of MEMα supplemented with 10% FBS. The culture supernatant was subsequently collected, cleared of cells by centrifugation at 220 × g for 5 min, and used as conditioned medium for injection together with shRNA-expressing MC3T3-E1 cells and matrigel into nude mice.

### RNA interference

MC3T3-E1 cells in 24-well plates were transduced with lentiviral particles encoding POSTN (sc-29315-V, Santa Cruz Biotechnology, Santa Cruz, CA) or scrambled control (sc-108080, Santa Cruz Biotechnology) shRNAs. Cells stably expressing the shRNAs were selected by culture in the presence of puromycin.

### Microarray analysis

Samples were processed for microarray analysis at the Core Instrumentation Facility of the School of Medicine, Keio University. Total RNA was isolated from tissue derived from the patient’s primary (n = 3) and metastatic (n = 2) lesions using the Trizol reagent (Invitrogen) and was further purified with the use of the RNeasy Mini Kit (Qiagen, Hilden, Germany). Biotinylated cRNA was synthesized from the total RNA and subjected to hybridization with a Human Genome U133 Plus 2.0 array (Affymetrix Inc. Santa Clara, CA). For identification of genes overexpressed in the metastatic lesion, the raw expression data for each probe set were generated with the use of GeneSpring GX Software (Agilent Technologies, Santa Clara, CA). Probe sets identified as being expressed (present call) in all primary and metastatic lesions samples were analyzed. The metastasis/primary expression ratio for each probe was calculated after normalization and genes with a >4.0-fold change in expression on average were selected. Microarray data are available in the GEO database (http://www.ncbi.nlm.nih.gov/geo) under the accession number GSE62837.

### Bioinformatics analysis

The list of genes showing a >4-fold increase in expression in the metastatic lesion compared with the primary lesion was uploaded in Database for Annotation, Visualization and Integrated Discovery (DAVID; version 6.7; http://david.abcc.ncifcrf.gov), and gene clustering analysis was performed with the following settings: similarity term overlap, 7; similarity threshold, 0.50; initial group membership, 4; final group membership, 4; and multiple linkage threshold, 0.50. The probability (*P*) value was based on a modified Fisher’s exact test in the DAVID system (corresponding to the one-tailed Fisher’s exact probability value).

### Animal studies

Balb/c nude mice were obtained from Sankyo Labo Service Corporation (Tokyo, Japan) and maintained in the animal facility at Keio University. In each procedure, male mice at 6–8 weeks of age were anesthetized with 4.0% sevoflurane in oxygen. For analysis of gene expression in wound tissue, five full-thickness excision wounds were inflicted on the dorsal side of the animals with a 5-mm sterile disposable biopsy punch (Maruho Industries, Osaka, Japan). The excised tissue (control) and wound tissues subsequently excised with a 2-mm margin at 4, 7, 10, 13, and 23 days after injury were analyzed. For the wound metastasis assay, B16-BL6 cells (2.0 × 10^5^) were transplanted in the left hind footpad of mice at day 0, and a full-thickness skin wound was inflicted on the left thigh with the use of the 5-mm biopsy punch on day 3. Instead of wounding, some mice received a subcutaneous injection of 1.5 × 10^6^ MC3T3-E1 cells (stably expressing POSTN or control shRNAs) mixed with 75 μl of MC3T3-E1 cell–conditioned medium and 75 μl of matrigel (BD Matrigel Matrix Growth Factor Reduced, BD Biosciences, Franklin Lakes, NJ) in the left thigh at day 7. All mice were killed by cervical dislocation after having been anesthetized by 4.0% sevoflurane in oxygen at day 23. Tissue was removed from the wound or injection site at the left thigh, fixed, sectioned, and stained with H&E, and the longest diameter of the metastatic lesion was measured by microscopy.

### Quantitative RT-PCR analysis

Total RNA of the cell lines and wound tissues from Balb/c nude mice were extracted with the use of an RNeasy Mini Kit (Qiagen). The RNA isolated from cell lines, murine wound tissue, and tissue of the melanoma patient was subjected to reverse transcription with the use of a Prime Script RT-PCR Kit (Takara Bio, Otsu, Japan) followed by quantitative PCR analysis with the use of SYBR Green and a Thermal Cycler Dice Real Time System (Takara Bio), as previously described [15]. Gene expression was normalized by the corresponding amount of *Gapdh* or *GAPDH* mRNA. The sequences of the PCR primers (forward and reverse, respectively) were as follows: mouse *Postn*,

5′-TGCTGCCCTGGCTATATGAG-3′ and 5′-GTAGTGGCTCCCACAATGCC-3′; mouse *Col1a1*, 5′-GAGCCCTCGCTTCCGTACTC-3′ and 5′-TGTTCCCTACTCAGCCGTCTGT-3′; mouse *Fn1*, 5′-TGCACGATGATATGGAGAGC-3′ and 5′-TGGGTGTCACCTGACTGAAC-3′; mouse *Tgfb*, 5′-TTGCTTCAGCTCCACAGAGA-3′ and 5′-TGGTTGTAGAGGGCAAGGAC-3′; mouse *Gapdh*, 5′-GTGAAGGTCGGTGTGAACG-3′ and 5′-GACCATGTAGTTGAGGTCAATG-3′; human *POSTN*, 5′-GTTTGTTCGTGGTAGCACCT-3′ and 5′-TGTTGGCTTGCAACTTCCTCAC-3′; human *COL1A1*, 5′-GTCACTGTCGATGGCTGC-3′ and 5′-CGTCGAAGCCGAATTCCTG-3′; human *FN1*, 5′-CCAGTCCTACAACCAGTATTCTC-3′ and 5′-CTTCTCTGTCAGCCTGTACATC-3′; and human *GAPDH*, 5′-GCACCGTCAAGGCTGAGAAC-3′ and 5′-TGGTGAAGACGCCAGTGGA-3′.

### Immunohistochemistry and immunohistofluorescence analysis

Tissue was fixed with 4% paraformaldehyde, embedded in paraffin, and sectioned at a thickness of 4 μm. For immunohistochemistry, antibody binding was visualized with the universal immuno-alkaline-phosphatase polymer method (N-Histofine Simple Stain Multi, Nichirei Bioscience, Tokyo, Japan). Sections were stained with a mouse mAb to POSTN (clone no. SS19C) [16], a mouse mAb to COL-I (Abcam, Cambridge, UK), a mouse mAb to FN (Abcam), a rabbit mAb to Melan-A (Abcam), and a rabbit polyclonal Ab to S100 (Dako, Glostrup, Denmark). Sections were counterstained with hematoxylin to visualize cell nuclei. For immunohistofluorescence analysis, binding of the antibodies to POSTN, COL-I, and FN was visualized with Alexa Fluor 594-labeled goat antibodies to mouse IgG (Invitrogen) and that of those to Melan-A was visualized with Alexa Fluor 488-labeled goat antibodies to rabbit IgG (Invitrogen).

### Immunoblot analysis

Normal mouse skin tissue and mouse wound tissue 7 and 23 days post wounding were lysed in radioimmunoprecipitation (RIPA) buffer supplemented with a phosphatase inhibitor (PhosSTOP, Roche). The lysates were incubated for 20 min on ice and centrifuged at 20,000 × *g* for 15 min at 4°C. The supernatants were denatured at 95°C for 5 min in SDS sample buffer, consisting of 62.5 mmol/L Tris (pH 6.8), 10% glycerol, 2% SDS, 5% 2-mercaptoethanol, and 0.001% bromophenol blue. Samples were separated by SDS–PAGE, and proteins were transferred onto a PDVF membrane (Bio-Rad, Munich, Germany). Membranes were incubated for 30 min at room temperature in blocking buffer consisting of 5% nonfat dry milk in phosphate-buffered saline (PBS) with 0.05% Tween-20, followed by an appropriate dilution of anti-POSTN Ab (Abcam) or anti-α-tubulin (Sigma) primary antibody overnight at 4°C. Immune complexes were detected with horseradish peroxidase-conjugated secondary antibodies (GE Healthcare, Piscataway, NJ), a chemiluminescence detection system (Perkin-Elmer, Waltham, MA), and a LAS-3000 instrument (Fujifilm, Tokyo, Japan)

### Scratch wound healing assay

The wells of a 96-well flat-bottom plate were incubated overnight at 4°C with recombinant human POSTN (R&D Systems, Minneapolis, MN), mouse POSTN (R&D Systems), human COL-I (BD Biosciences), or human FN (Roche, Mannheim, Germany), each at 10 μg/ml. Noncoated wells served as controls. The plates were washed twice with PBS, after which B16-BL6 cells (2.0 × 10^4^) suspended in 100 μl of serum-free medium were added to each well coated with mouse POSTN, human COL-I, human FN, or noncoated wells. Alternatively, MeWo cells (5.0 × 10^4^) in 100 μl of serum-free medium were added to each well coated with human POSTN, COL-I, FN, or noncoated wells. All cells were incubated at 37°C for 36 h in order to grown to confluence. Subsequently, an artificial wound was generated by dragging a 200-μl pipette tip through the cell monolayer and cells were allowed to grown under 37°C for further 36 h. In some experiments, 10 μg/ml of anti–mouse integrin αv Ab (BioLegend, San Diego, CA) or 10 μg/ml of rat IgG1 isotype control Ab (eBioscience, San Diego, CA) was administered to wells with B16-BL6 cells soon after the artificial wound was generated. The cells were examined with the use of an inverted phase-contract microscope and photographed at baseline (0 h) and 36 h after wounding for determination of the extent of wound closure. The migration ability of the tumor cells was expressed as closed width/scratch (%).

### Transwell migration assay

The transwell migration assay was performed according to a modified version of a previously described method [13]. The lower surface of Falcon cell culture inserts (8 μm, BD Biosciences) was coated with 50 μl of human FN (20 μg/ml) to support cell attachment, and the upper surface was coated with 50 μl of mouse or human POSTN, human POSTN lacking the C-terminus (POSTN-ΔC) (Biovendor, Heidelberg, Germany), COL-I, or FN (each at 40 μg/ml). Noncoated wells served as a control. For measurement of spontaneous cell migration, B16-BL6 or MeWo cells (2.0 × 10^4^ per well) suspended in 200 μl of serum-free medium were added to the upper surface of each insert, and the lower chamber was filled with 800 μl of serum-free medium. In some experiments, B16-BL6 cells were incubated with 10 μg/ml of anti-mouse integrin αv Ab or 10 μg/ml of rat IgG1 isotype control Ab for 2 h and cells were then added to the upper surface of the inserts. After incubation for 12 h at 37°C, cells on the upper surface of each filter were removed with a cotton swab, and cells on the lower surface of the filters were fixed with 100% methanol and stained with Diff-Quik (Sysmex Corporation, Kobe, Japan). The migration ability of the tumor cells was expressed as the mean number of cells per field, with analysis of five fields in total.

### Adhesion assay

B16-BL6 and MeWo cells (2.0 × 10^4^ per well) in 100 μl of serum-free medium were transferred to 96-well flat-bottom plates coated with POSTN, POSTN-ΔC, COL-I, or FN as described above for the scratch wound assay. Noncoated wells served as controls. The cells were incubated for 90 min at 37°C, the wells were washed twice with PBS, and attached cells were assayed for each well with the use of a Cell Titer-Glo luminescence cell viability kit (Promega, Madison, WI). In some experiments, B16-BL6 cells were incubated with 10 μg/ml of anti–mouse integrin αv Ab or 10 μg/ml of rat IgG1 isotype control Ab for 2 h and cells were then transferred to 96-well flat-bottom plates. The resulting values are expressed as percentages relative to the control (100%).

### Proliferation assay

B16-BL6 and MeWo cells suspended in 100 μl of medium supplemented with 10% FBS were seeded at a density of 2.0 × 10^3^ cells per well in 96-well flat-bottom plates coated with POSTN, COL-I, or FN as described above. Noncoated wells served as controls. The cells were cultured for 72 h, washed twice with PBS, and assayed for cell proliferation with the use of a Cell Titer-Glo luminescence cell viability kit (Promega). Data are expressed relative to the values of cells cultured for 12 h.

### Statistics

Data are presented as means ± SD and were analyzed with the unpaired Student’s *t* test. A *P* value of <0.05 was considered statistically significant.

### Ethics Statement

All animal studies were performed according to protocols approved by the Ethics Committee of Keio University. All human studies were approved by the Institutional Review Board of Keio University. The study has fulfilled the Declaration of Helsinki Principles. All patients gave written informed consent.

## Results

### Acral lentiginous melanoma patient with wound site metastasis

**Fig 1:**
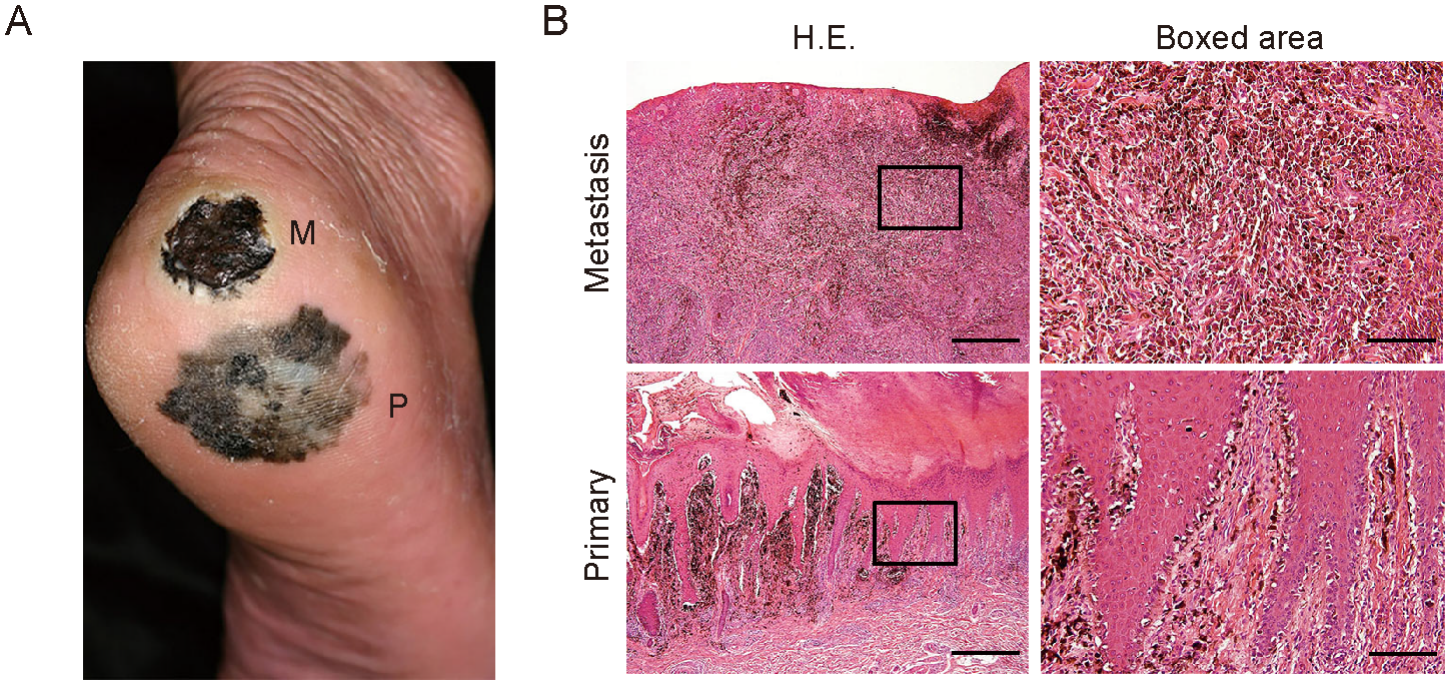
Clinical manifestation and histopathology of the acral lentiginous melanoma patient with wound site metastasis. (A) Clinical presentation of the right foot of the patient. P, primary lesion; M, metastatic lesion. (B) H&E staining of the primary and metastatic lesions of the patient. The boxed regions are shown at higher magnification in the right panels. Bars: 500 μm (left) or 100 μm (right).

A 61-year-old man presented with a 2-year history of a blue–black patch that measured 37 by 28 mm and had an irregular border on the sole of his right foot. He also reported a 9-month history of an ulcerated black plaque with a diameter of 25 mm on the right heel that began to develop 1 month after a trauma, enlarged rapidly, and formed an ulcer within 3 months (Fig 1A). A wide local excision was performed with a 2-cm margin. Histopathologic examination of the primary lesion revealed proliferation of atypical melanocytes in the basal layer and superficial dermis (Fig 1B). The tumor thickness was 1.6 mm, and tumor cells were positive for S100, gp100, and Melan-A (data not shown). On the basis of these pathological findings, a diagnosis of acral lentiginous melanoma was confirmed. Despite its smaller size relative to the primary lesion, the secondary lesion was indeed ulcerated and its histopathology revealed diffuse infiltration of melanoma cells throughout the dermis (Fig 1B), suggesting aggressive biological behavior.

### POSTN, COL-I, and FN were highly expressed in the stroma of the wound metastatic lesion of melanoma

**Fig 2:**
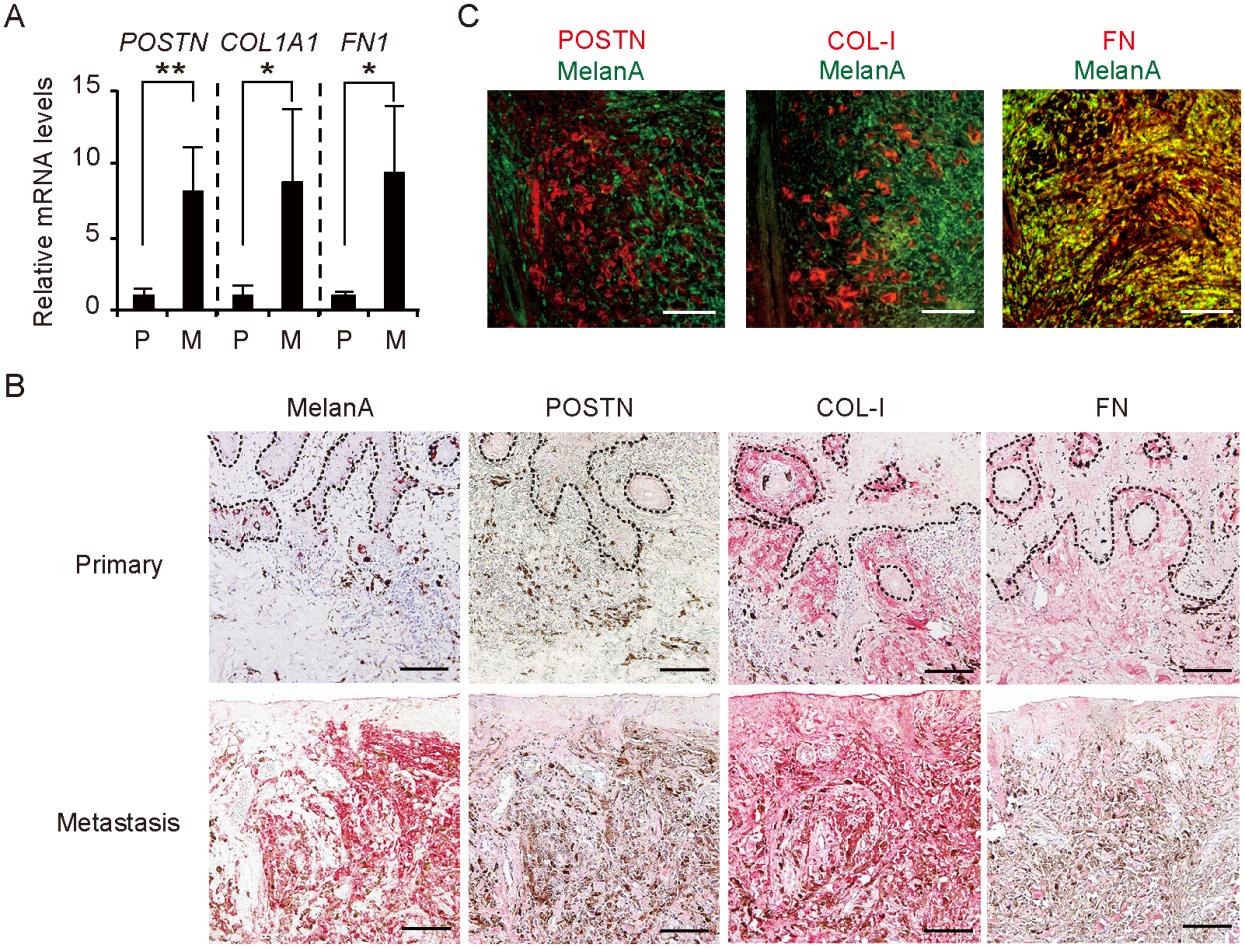
Overexpression of POSTN, COL-1, and FN in the stroma of wound metastasis in a melanoma patient. (A) Quantitative RT-PCR analysis of *POSTN*, *COL1A1*, and *FN1* mRNA expression in the primary (n = 3) and metastatic (n = 2) lesions. Data are means ± SD for triplicate samples from one of three representative experiments. *P < 0.05, **P < 0.005. (B) Immunohistochemical staining of the primary and metastatic lesions with antibodies to Melan-A, POSTN, COL-I, and FN. Dashed lines indicate the basement membrane zone. (C) Immunofluorescence analysis of the lower dermis of the metastatic lesion with antibodies to Melan-A (green) as well as those to POSTN, COL-I, or FN (red). FN showed colocalization (yellow) with Melan-A (melanoma cell marker), whereas POSTN and COL-I were localized only in the stroma. (B, C) Bars = 100 μm.

To explore the molecular mechanism underlying the promotion of wound metastasis in melanoma, we performed microarray analysis of the metastatic lesions (n = 2) and the primary lesions (n = 3) of the patient. Analysis of 54,675 probe sets revealed that 539 genes exhibited a >4-fold increase in expression level in the metastatic lesion compared with the primary lesion (S1 Table). Functional classification analysis with the use of DAVID software showed that these genes could be divided into 46 clusters on the basis of their enrichment scores [17, 18]. The three clusters with high enrichment scores (>3.0) comprised a total of 78 genes related to cell motion, pigmentation, and skeletal system development (S2 Table). To select molecules for further investigation, we applied the following criteria to these 78 genes: (i) the expression signal for probe sets was >20 in all samples (given that probe sets with low signals are not measured reliably); (ii) the gene is expressed predominantly in the stroma; and (iii) the gene is relevant to wound healing. We found that *POSTN*, collagen type 1 alpha 1 (*COL1A1*), and fibronectin 1 (*FN1*) genes met all these criteria. Quantitative RT-PCR analysis confirmed that the abundance of mRNAs for these three genes was significantly increased in the metastatic lesion compared with the primary lesion (Fig 2A).

To examine the expression and distribution of POSTN, COL-I, and FN in the tissue specimens, we performed immunohistochemical analysis with the use of alkaline-phosphatase polymer (red) to enable immunoreactivity to be distinguished from melanin. The relative area occupied by POSTN, COL-I, or FN was larger in the metastatic lesion than in the primary lesion (Fig 2B). Of note, the distribution of POSTN differed between the primary and metastatic lesions, whereas both COL-I and FN were localized to the dermis of both lesions. POSTN was thus detected at low levels along the basement membrane in the primary lesion, as in normal skin, whereas it was diffusely distributed in the dermis of the metastatic lesion. Immunofluorescence analysis of the metastatic lesion revealed that FN was expressed in both stromal and melanoma (Melan-A–positive) cells, whereas POSTN and COL-I were localized only in stromal cells (Fig 2C). We also examined vessels in the dermis of the metastatic lesion and found that POSTN, COL-I, and FN were all expressed in the dermal matrix interfacing with endothelial cells (S1 Fig). Together, these observations suggested that POSTN, COL-I, and FN components of fibrillar networks were overexpressed in the dermal matrix surrounding melanoma cell nests as well as in perivascular regions of the metastatic lesion.

### Injury promoted metastasis of melanoma to the wound region in a mouse model

**Fig 3:**
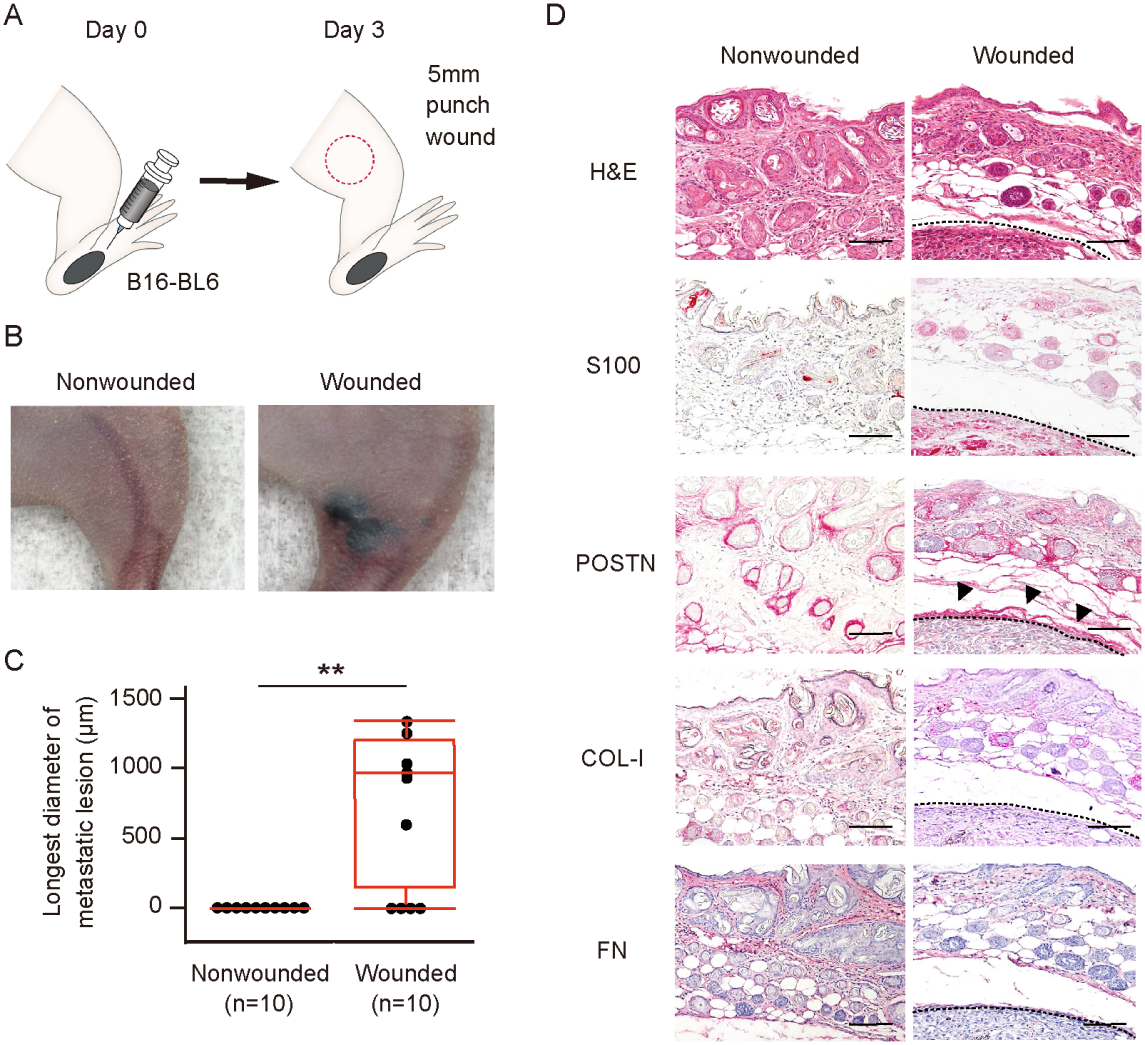
Wound healing process predisposes to metastasis of melanoma *in vivo*. (A) Schematic representation of the wound metastasis model. B16-BL6 cells were transplanted into the left hind footpad of a Balb/c nude mouse on day 0, and a 5-mm full-thickness skin wound was inflicted on the left thigh (red circle) on day 3. (B, C) Representative images of (B) and longest diameter of subcutaneous metastasis at (C) the left thigh for wounded and nonwounded (control) mice (n = 10 each) on day 23. Quantitative data are presented as a box-and-whisker plot. **P < 0.005. (D) Representative immunohistochemical staining of S100 (melanoma cell marker), POSTN, COL-I, and FN as well as H&E staining for the left thigh of wounded and nonwounded mice at day 23. Arrowheads indicate POSTN expression surrounding a melanoma tumor cell nest. Dashed lines indicate the periphery of tumor cell nests. Bars = 100 μm.

To provide further mechanistic insight into wound metastasis, we recapitulated the process in an animal model. We transplanted murine melanoma B16-BL6 cells into the footpad of Balb/c nude mice on day 0 and inflicted a wound to the thigh on day 3 (Fig 3A). On day 23, 6 of 10 wounded mice manifested subcutaneous metastasis at the wound region, whereas no such metastasis was detected in any of the 10 nonwounded mice (Fig 3B and 3C). Immunohistochemical analysis of the metastatic lesions revealed that POSTN was highly expressed at the periphery of the melanoma tumor cell nests. In contrast, COL-I was not detected and FN was expressed only at a low level (Fig 3D). These results suggested that, among these three fibrillar network components, POSTN is most likely to play a role in wound metastasis in this model.

### POSTN expression is upregulated and sustained following wound healing

**Fig 4:**
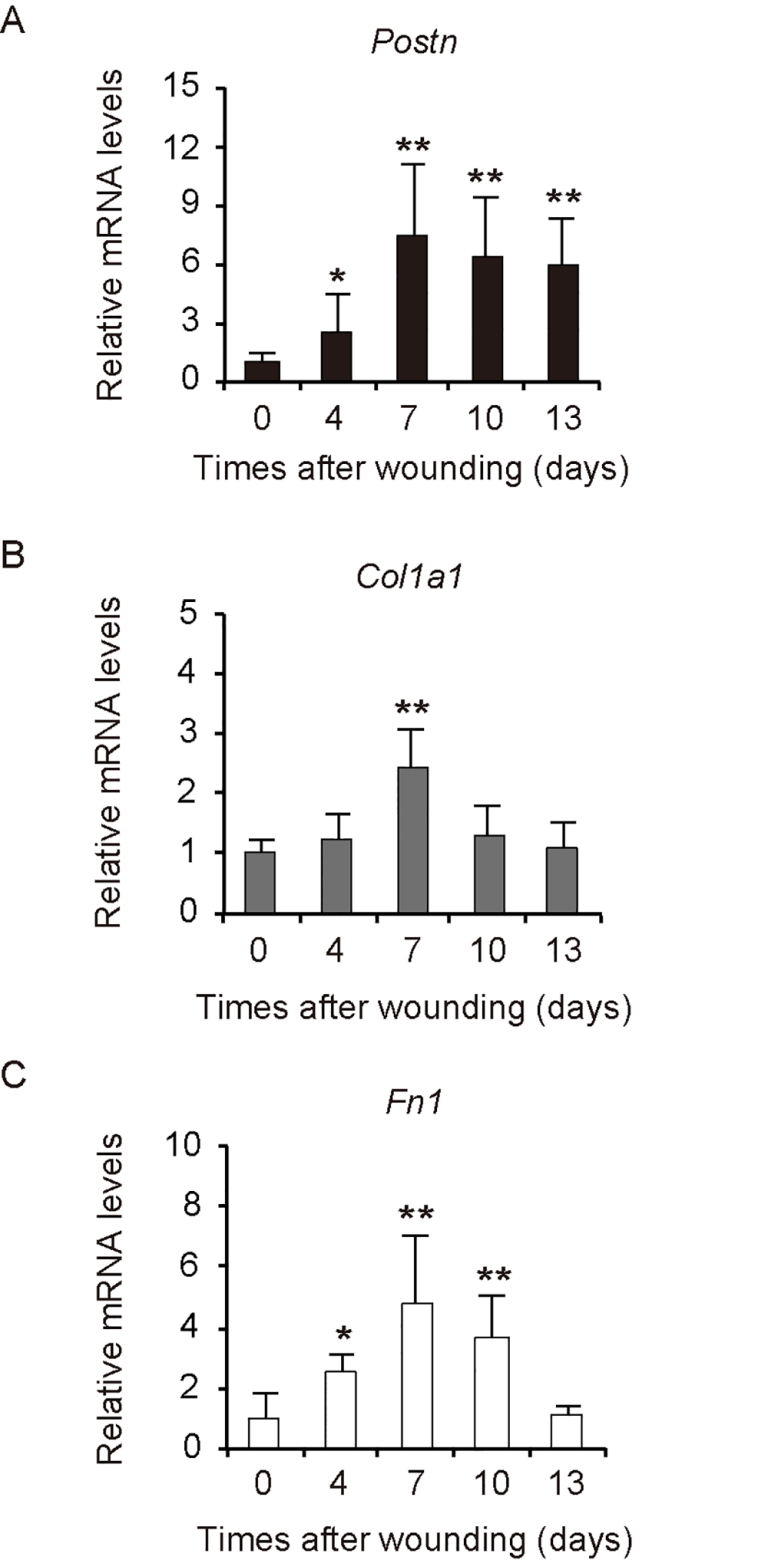
Wound healing process increases expression of *Postn*, *Col1a1*, and *Fn1* in a mouse model. (A-C) Quantitative RT-PCR analysis of (A) *Postn*, (B) *Col1a1*, and (C) *Fn1* mRNA expression in wound tissue removed from nude mice (n = 6) at 4, 7, 10, or 13 days after injury. Intact skin (0 days) was examined as a control. Data are means ± SD from one experiment representative of three independent experiments. *P < 0.05, **P < 0.005 *vs*. intact skin.

We further examined the expression of *Postn*, *Col1a1*, and *Fn1* mRNA during wound healing in the nude mice. Quantitative RT-PCR analysis revealed that the abundance of *Postn* mRNA was increased at the wound site 4 days after injury and it was still significantly greater than that in the normal skin by day 13 (Fig 4A). In contrast, *Col1a1* mRNA increased at day 7 and declined to a level similar to that in the normal skin by day 10 (Fig 4B). The amount of *Fn1* mRNA increased at day 4 but had also declined by day 13 (Fig 4C). Among these three ECM proteins, POSTN therefore appeared to be involved in the wound healing process for the longest period. Furthermore, we examined the expression of *Postn* mRNA on day 23 after wounding and found that *Postn* mRNA expression decreased to a level similar to normal skin. Similar results were obtained with the analysis of *Col1a1* and *Fn1* mRNA on day 23 after wounding (Panel A in S2 Fig). In addition, we performed western blot analysis to relatively quantitate POSTN expression in normal skin and wound tissue at 7 days and 23 days post wounding. The POSTN expression reflected the results of qRT-PCR analysis at these time points, showing higher expression in the wound of day 7 than that of day 23 and normal skin (Panel B in S2 Fig).

### POSTN attenuated the adhesion and promoted migration of melanoma cells *in vitro*

**Fig 5:**
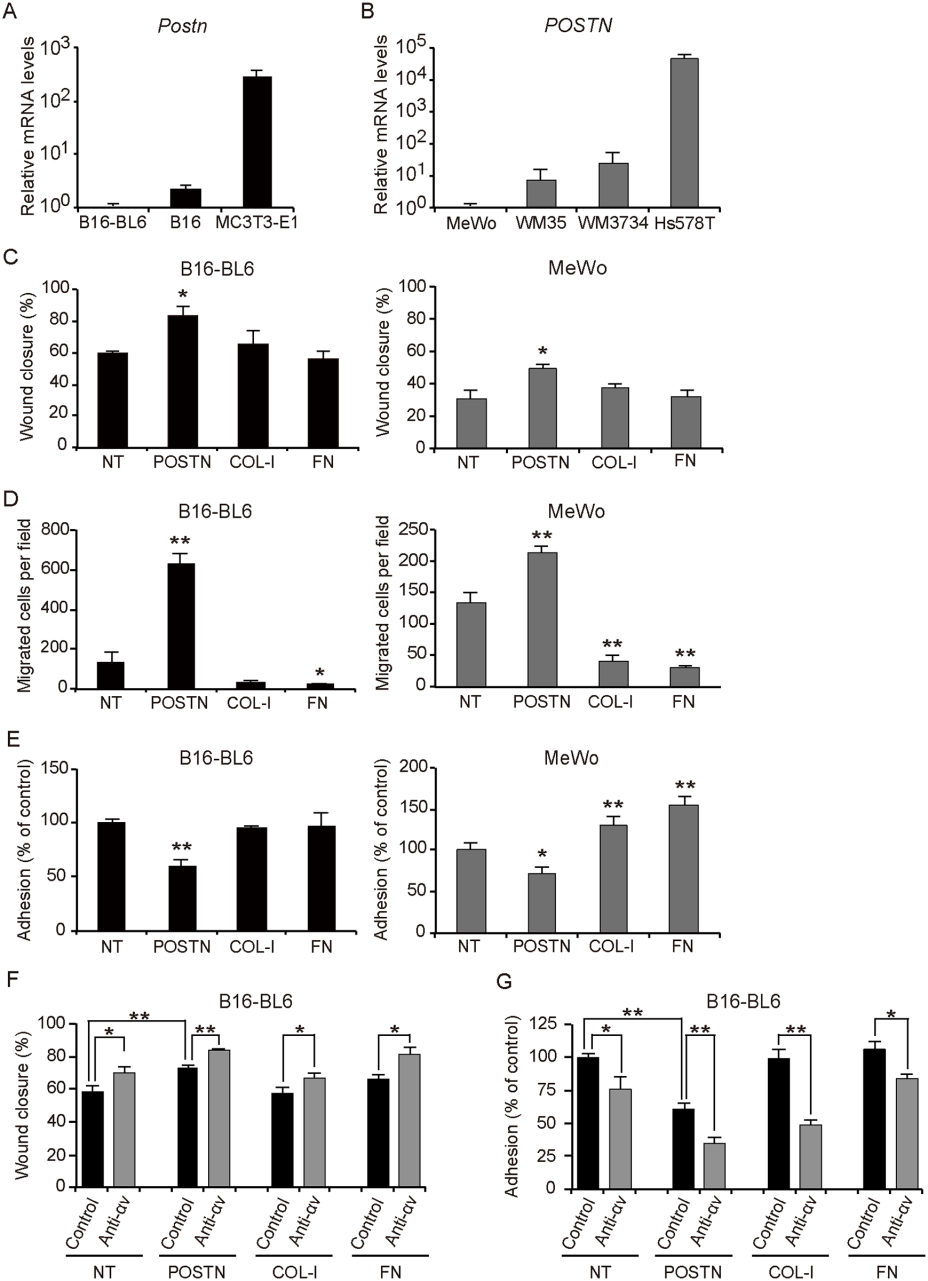
POSTN attenuates cell adhesion and promotes migration of melanoma cells *in vitro*. (A, B) Quantitative RT-PCR analysis of (A) *Postn* mRNA expression in B16, B16-BL6, and MC3T3-E1, and (B) *POSTN* mRNA expression in the human melanoma cell lines MeWo, WM35, and WM3734 as well as in the human breast cancer cell line Hs578T. Data in (A, B) are means ± SD for triplicate experiments. (C–E) Scratch wound healing assay (C), transwell migration assay (D), and adhesion assay (E) for B16-BL6 and MeWo cells cultured in wells or on culture inserts coated (or not, NT) with POSTN, COL-I, or FN. (F-G) Scratch wound healing assay (F) and adhesion assay (G) for B16-BL6 cells cultured in wells or on culture inserts coated (or not, NT) with POSTN, COL-I, or FN with anti-integrin αv Ab or control Ab. Data in (C-G) represent mean values of triplicates ± SD. Similar results were obtained in three separate experiments. *P < 0.05, **P < 0.005 *vs*. NT or control Ab.

In a mouse breast cancer model, it was recently shown that stromal POSTN in the lung promotes metastatic colonization by interacting with breast cancer stem cells and serving as a metastatic niche [9]. Given the prolonged induction of *Postn* mRNA during wound healing and the high expression level of POSTN in the region surrounding melanoma cell nests in our mouse model, we subsequently focused on the role of this protein in wound metastasis. To evaluate the effects of POSTN in the metastatic tumor microenvironment, we studied B16-BL6 and the human melanoma cell line MeWo, both of which contain only small amounts of *Postn and POSTN* mRNA, respectively (Fig 5A and 5B). We first examined the motility of these melanoma cells in a scratch wound healing assay. The coating of POSTN on the plate significantly increased the rate of wound closure by B16-BL6 and MeWo cells, whereas COL-I and FN had no such effect (Fig 5C). A transwell migration assay also revealed that filters coated with POSTN promoted the migration of both B16-BL6 and MeWo cells, whereas those coated with COL-I or FN did not (Fig 5D). Furthermore, an adhesion assay showed that POSTN coated wells significantly attenuated the adhesion of B16-BL6 cells compared with noncoated wells (Fig 5E). POSTN also significantly inhibited the adhesion of MeWo cells, whereas COL-I and FN enhanced the adhesion of these cells (Fig 5E). Given that POSTN produced by cancer-associated fibroblasts was recently shown to promote proliferation of melanoma cells by activating MAPK signaling [19], we also examined the possible effects of POSTN, COL-I, and FN on melanoma cell proliferation. However, both B16-BL6 and MeWo cells manifested similar growth rates in the absence or presence of POSTN, COL-I, or FN (S3 Fig). Together, these findings indicated that POSTN attenuates cell adhesion and promotes the migration of melanoma cells.

Integrin αvβ3 and αvβ5 are known receptors for POSTN, and it was also reported that B16-BL6 cells express integrin αvβ3, and MeWo cells express integrin αvβ3 and αvβ5 [19, 20]. To assess the contribution of integrin αv to the migration and anti-adhesion effects of B16-BL6 cells, we administrated a neutralizing anti-αv integrin Ab and performed a scratch wound healing assay. The anti-integrin αv Ab significantly increased the rate of wound closure by B16-BL6 on POSTN coated plates compared with control Ab. Interestingly, a similar result was obtained in uncoated, COL-1 or FN coated plates (Fig 5F). A transwell migration assay also revealed that administration of anti-integrin αv Ab promoted the migration of B16-BL6 cells than those treated with control Ab in filters coated with POSTN (S4 Fig). In addition, an adhesion assay showed that anti-integrin αv Ab treatment significantly attenuated the adhesion of B16-BL6 cells compared with control Ab treatment of POSTN coated wells. Furthermore, a similar result was obtained in uncoated, COL-1 or FN coated wells suggesting that integrin αv is not associated with the anti-adhesive properties of full-length POSTN (Fig 5G).

### Inhibition of microenvironmental POSTN reduced the incidence of melanoma metastasis

**Fig 6:**
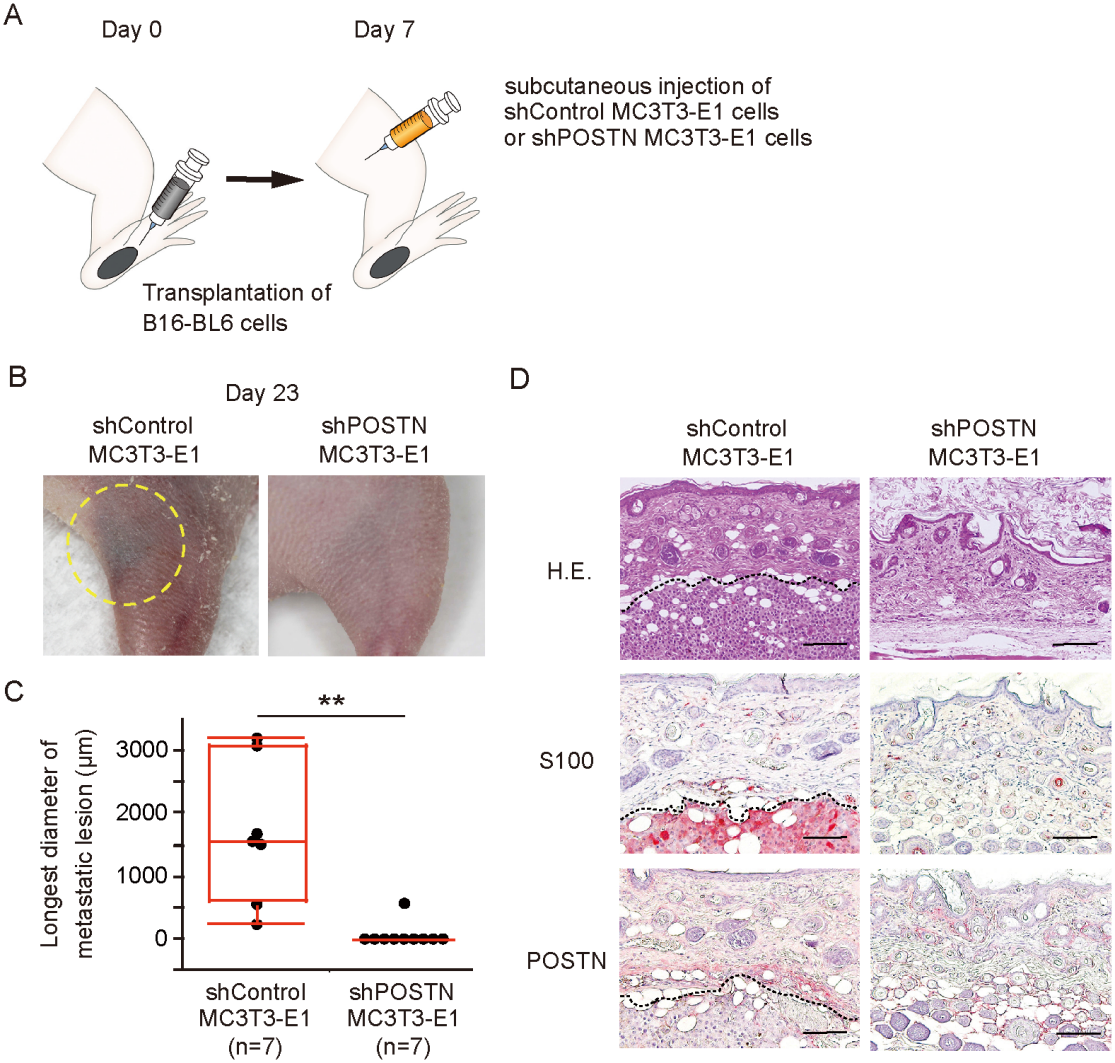
Inhibition of POSTN expression in microenvironment reduces incidence of melanoma wound metastasis. (A) Experimental protocol for *in vivo* assay of metastasis. B16-BL6 cells were transplanted into the left hind footpad of nude mice on day 0, and MC3T3-E1 cells stably expressing control or POSTN shRNAs were injected subcutaneously into the left thigh on day 7. (B, C) Representative images (yellow circle indicates subcutaneous metastasis) of (B) and longest diameter of subcutaneous metastasis at (C) the left thigh on day 23 for mice (n = 7 each) treated as in (A). Quantitative data are presented as a box-and-whisker plot. **P < 0.005. (D) Representative immunohistochemical staining of S100 and POSTN as well as H&E staining at day 23 for the left thigh of mice treated as in (A). Arrowheads indicate POSTN expression in the region surrounding a melanoma tumor cell nest. Dashed lines indicate the periphery of tumor cell nest. Bars = 100 μm.

Finally, we attempted to confirm a role for POSTN in the colonization of melanoma cells *in vivo*. MC3T3-E1 cell is a murine osteoblastic cell line that stably secretes large amounts of POSTN. We established MC3T3-E1 cells that stably express either a scrambled (control) shRNA (shControl MC3T3-E1 cells) or a POSTN shRNA (shPOSTN MC3T3-E1 cells). The abundance of *Postn* mRNA was significantly reduced in shPOSTN MC3T3-E1 cells compared with shControl MC3T3-E1 cells (S5 Fig). To evaluate the relation between POSTN expression and the ability to attract melanoma cells, we injected shControl or shPOSTN MC3T3-E1 cells mixed with shControl or shPOSTN MC3T3-E1 cell-conditioned medium, which was added for the purpose of introducing a peak POSTN expression similar to endogenous POSTN expression during the wound healing process, and matrigel into the thigh 7 days after injection of B16-BL6 melanoma cells into the footpad of nude mice (Fig 6A). The incidence of subcutaneous metastasis at the MC3T3-E1 cell injection region (1 of 7 *vs*. 7 of 7) and the longest diameter of the metastatic lesion at day 23 were significantly reduced in mice injected with shPOSTN MC3T3-E1 cells compared with those injected with shControl MC3T3-E1 cells (Fig 6B and 6C). Immunohistochemical staining revealed POSTN expression in the tissue surrounding melanoma tumor cell nests at the metastatic lesions of mice injected with shControl MC3T3-E1 cells (Fig 6D). Hence, these results indicated that POSTN promotes metastasis of melanoma.

## Discussion

In this study, we aimed to explore the factors relevant to wound healing process that promote wound metastasis of melanoma. Metastasis is a multistage process involving tumor cell intravasation, transit in the vessels, extravasation and growth at a new site [21]. As circulating melanoma cells have been reported to be detected even from stage I melanoma [22], we speculated that the microenvironment of wound healing contributed to promote extravasation or proliferation of circulating melanoma cells, leading to promotion of their colonization. By comparing gene expression in the primary and wound metastatic lesion of the melanoma patient, we found that among genes related to wound healing, genes of ECM proteins, including *POSTN*, *COL1A1*, and *FN1*, were overexpressed in the wound metastatic lesion. Furthermore, we succeeded to recapitulate the process of wound-induced metastasis of melanoma cells in a mouse model.

ECM provides structural as well as functional integrity to connective tissue [5]. COL-I and FN form fibrils that contribute to cell adhesion [23]. In addition, FN promotes the migration of fibroblasts into injured tissue [23], whereas POSTN promotes the migration of fibroblasts as well as the proliferation of fibroblasts and keratinocytes during wound healing [10, 12]. Given that wound healing and tumor progression both involve cell proliferation and inflammation [24, 25], we investigated whether POSTN, COL-I, and FN also influence tumor cell function. We found that COL-I and FN maintained or enhanced melanoma cell adhesion, whereas POSTN attenuated adhesion and enhanced the migration of melanoma cells *in vitro*. POSTN may thus regulate tumor cell adhesion to COL-I and FN and thereby promote the extravasation of these cells to wound sites, a process that requires sequential cell attachment and detachment [13]. Indeed, downregulation of POSTN reduced the incidence and extent of metastasis *in vivo*, implicating POSTN as a major niche component for metastasis of melanoma to wound sites.

We showed that all mice receiving subcutaneous injections of shControl MC3T3-E1 cells plus shControl MC3T3-E1 cell-conditioned medium developed metastasis at the injected region, whereas only 6 out of 10 mice manifested metastasis at the wound region. We analyzed the *Postn* mRNA expression of wound tissues of 7 mice at day 7, the period that has been reported to be the peak of *Postn* mRNA expression during the wound healing process [11, 12], and found that the maximum expression level of *Postn* mRNA varied with each mouse (S6 Fig). Given that *Postn* mRNA expression correlated with the incidence of metastatic lesion formation in mice injected with MC3T3-E1 cells, the heterogeneity in the maximum expression level of *Postn* mRNA between wounded mice appears to be the basis for not all mice with wounds developing metastatic lesions at the wound site.

Recently it was reported that POSTN accelerates proliferation of several types of cancer cells, such as gastric cancer and melanoma cells [19, 26], while we found that B16-BL6 and MeWo cells manifested similar growth rates in the absence or presence of POSTN. A possible explanation for this discrepancy may be that researchers appear to have used lower concentrations of full-length POSTN or shorter forms of POSTN (not full-length POSTN) than in our study. One study performing a melanoma cell proliferation assay using full-length POSTN with same concentration did not show promotion of cell growth compared with other ECM proteins, which is in line with our results [13]. Thus, higher concentrations of full-length POSTN appear to predominantly affect adhesion and migration rather than proliferation of melanoma cells.

POSTN is a 90-kDa secreted protein, the protein structure of which is composed of an amino-terminal EMI domain, a tandem repeat of four FASI domains, and a carboxyl-terminal domain that includes a heparin binding site at its C-terminal end. POSTN is known to bind to integrins through its FAS1 domains, and its C-terminal domain gives rise to splice variants and contains proteolytic cleavage sites [8]. Given that integrin αv was not associated with the anti-adhesive properties of full-length POSTN and as POSTN lacking its C-terminus (POSTN-ΔC) was reported to function as an adhesive molecule [13], we performed an adhesion assay and a transwell migration assay with B16-BL6 cells using POSTN-ΔC. POSTN-ΔC significantly enhanced adhesion compared with full-length POSTN and showed similar adhesion to that of uncoated COL-1 or FN-coated wells (Panel A in S7 Fig). POSTN-ΔC also significantly suppressed migration compared with full-length POSTN (Panel B in S7 Fig). Similar results were obtained with MeWo cells. (data not shown). These results suggest that the C-terminus of POSTN is important for the anti-adhesive property.

Transforming growth factor-β (TGF-β) is a growth factor with a broad spectrum of effects during wound healing, including the induction of COL-I and FN expression [27]. It also induces the expression of POSTN in MC3T3-E1 cells [28], and coculture of melanoma cells with dermal fibroblasts was found to result in the induction of TGF-β expression in the melanoma cells and consequent upregulation of POSTN in the fibroblasts [19]. We examined *Tgfb* mRNA expression during wound healing in nude mice and found that the amount of *Tgfb* mRNA was significantly increased at 4 days after wounding and decreased to a level similar to normal skin at 23 days after wounding (S8 Fig), which was similar to the expression pattern of *Postn* mRNA (Fig 4A and Panel A in S2 Fig). These results suggest the possibility that TGF-β produced during the wound healing process contributes to the upregulating of POSTN expression.

Melanoma is characterized by its high metastatic potential. Tumor thickness and ulceration at the primary lesion correlate with each other as well as with the risk of metastasis [29]. We have now shown that the microenvironment of wound sites during the healing process, which resembles that of melanoma with ulceration, provides a premetastatic niche for melanoma cells and promotes their metastasis. Our findings shed light on the premetastatic niche for melanoma and suggest that targeting of POSTN warrants further investigation as a potential strategy for preventing metastasis.

## Supporting Information

S1 Fig Immunohistochemical analysis of POSTN, COL-1, and FN in perivascular regions of wound metastatic lesion. Arrowheads indicate vessels. Scale bars, 100 μm.
(TIF)

S2 Fig POSTN expression correlates with *Postn* mRNA expression during wound healing in nude mice. (A) The abundance of *Postn*, *Col1a1*, *and Fn1* mRNA at the wound site at 7 and 23 days post wounding (n = 6 each). Data represent mean values of triplicates ± SD. Similar results were obtained in two separate experiments. *P < 0.05, **P < 0.005 *vs*. intact skin. (B) Immunoblot analysis of POSTN expression in normal skin and tissues of wound site tissue at 7 and 23 days post wounding. α-Tubulin was analyzed as a loading control.
(TIF)

S3 Fig POSTN does not affect melanoma cell proliferation *in vitro*. Proliferation assay for MeWo (left) and B16-BL6 (right) cells cultured in wells coated (or not, NT) with POSTN, COL-I, or FN. Data represent mean values of triplicates ± SD. Similar results were obtained in three separate experiments.
(TIF)

S4 Fig Integrin αv is not related to the anti-adhesive properties of full-length POSTN. Transwell migration assay for B16-BL6 cells cultured in wells or on culture inserts coated with POSTN with anti-integrin αv Ab or control Ab. Data represent mean values of triplicates ± SD. Similar results were obtained in three separate experiments. *P < 0.05.
(TIF)

S5 Fig Depletion of periostin (*Postn*) mRNA in MC3T3-E1 cells by RNAi. The expression of *Postn* mRNA in MC3T3-E1 cells stably expressing control or POSTN shRNAs was measured by quantitative RT-PCR analysis. Data are means ± SD for triplicate experiments. **P < 0.005.
(TIF)

S6 Fig Maximum expression level of *Postn* mRNA varies in mice with wound healing process. Expression of *Postn* mRNA at the wound site 7 days post wounding in 7 mice (designated D7-a, D7-b, D7-c, D7-d, D7-e, D7-f, and D7-g) was measured by quantitative RT-PCR analysis. Data are means ± SD from one experiment representative of three independent experiments.
(TIF)

S7 Fig The C-terminus of full-length POSTN has a role in anti-adhesive properties. (A) Adhesion assay for B16-BL6 and cells cultured in wells or on culture inserts coated (or not, NT) with POSTN, POSTN-ΔC, COL-I, or FN. Data represent mean values of triplicates ± SD. Similar results were obtained in two separate experiments. **P < 0.005 *vs*. NT (B) Transwell migration assay for B16-BL6 cells cultured in wells or on culture inserts coated with POSTN or POSTN-ΔC. Similar results were obtained in two separate experiments. *P < 0.05.
(TIF)

S8 Fig Expression of *Tgfb* mRNA during wound healing in nude mice. The abundance of *Tgfb* mRNA at the wound site 4, 7, 10, 13, or 23 days post wounding (n = 6 each) was determined by quantitative RT-PCR analysis. Intact skin (baseline, 0 days) was examined as a control. Data are means ± SD from one experiment representative of two independent experiments. *P < 0.05, **P < 0.005 *vs*. intact skin.
(TIF)

S1 Table List of genes overexpressed over 4-fold in metastatic lesion compared with primary lesion of the patient. (XLSX)

S2 Table List of genes contained in clusters with high enrichment scores (>3) associated with the metastatic lesion of the patient as identified by gene clustering analysis with DAVID software. (XLSX)
