# Supplementary figures and images for: Periostin Is a Key Niche Component for Wound Metastasis of Melanoma
Source: PLoS One. 2015 Jun 17;10(6):e0129704. doi: 10.1371/journal.pone.0129704 (PMC4471156; doi:10.1371/journal.pone.0129704)

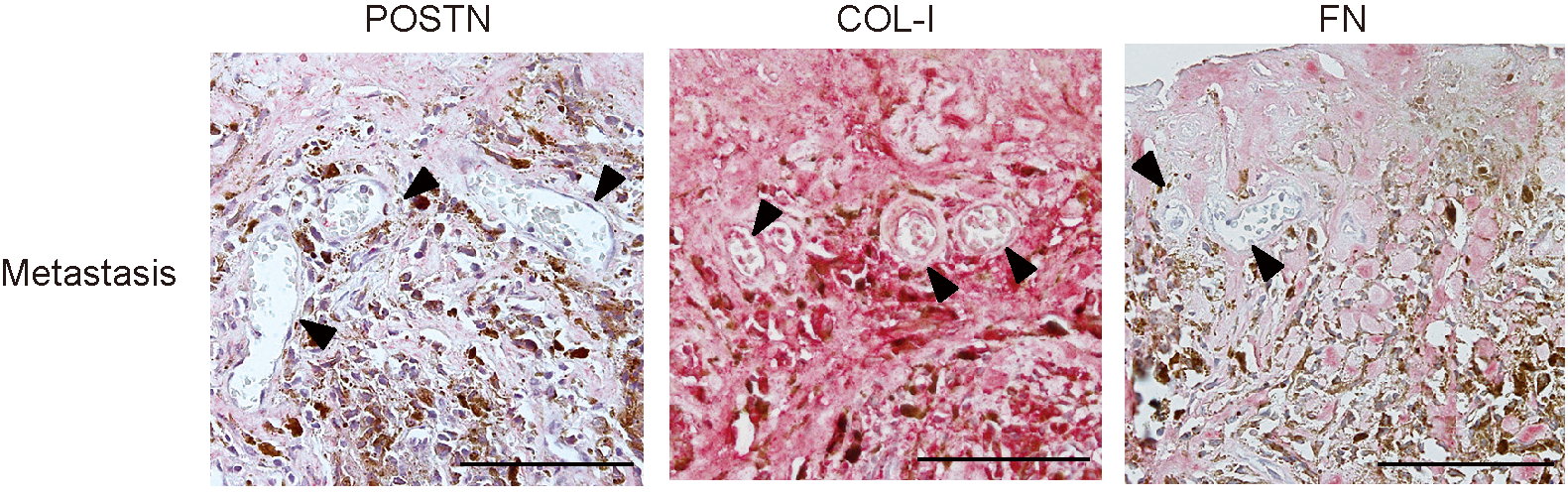

Supplement: S1 Fig — Arrowheads indicate vessels. Scale bars, 100 μm. (TIF) [file pone.0129704.s001.tif]

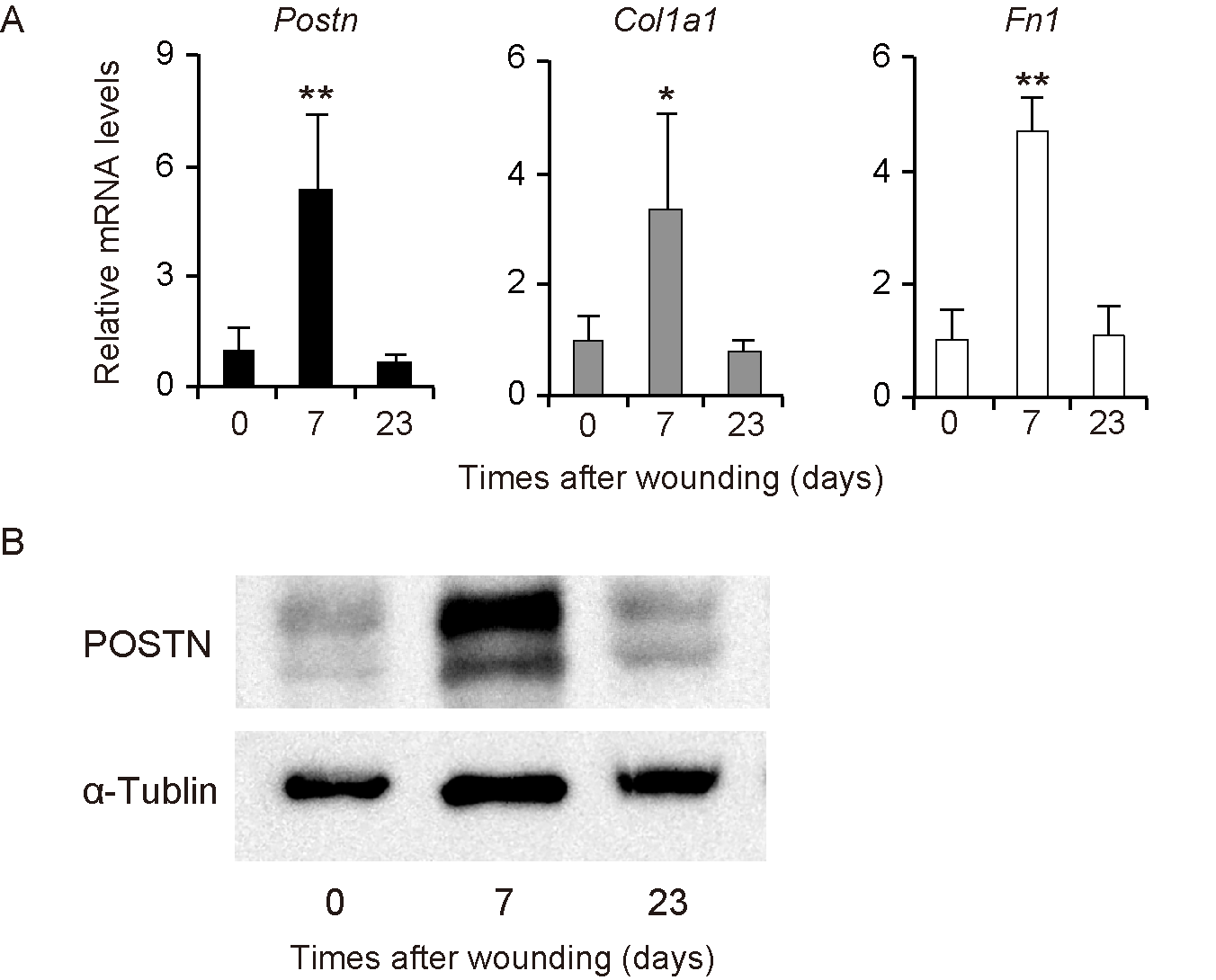

Supplement: S2 Fig — (A) The abundance of Postn, Col1a1, and Fn1 mRNA at the wound site at 7 and 23 days post wounding (n = 6 each). Data represent mean values of triplicates ± SD. Similar results were obtained in two separate experiments. *P < 0.05, **P < 0.005 vs. intact skin. (B) Immunoblot analysis of POSTN expression in normal skin and tissues of wound site tissue at 7 and 23 days post wounding. α-Tubulin was analyzed as a loading control. (TIF) [file pone.0129704.s002.tif]

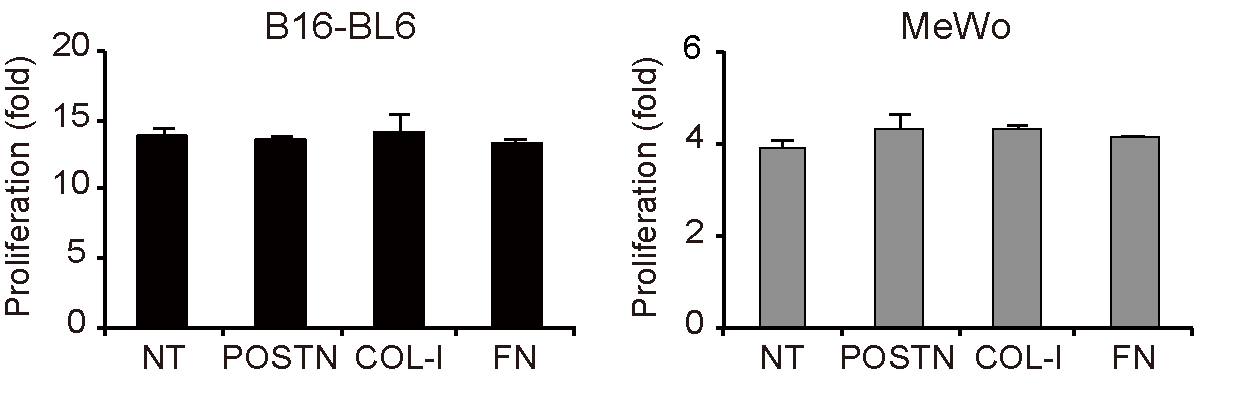

Supplement: S3 Fig — Proliferation assay for MeWo (left) and B16-BL6 (right) cells cultured in wells coated (or not, NT) with POSTN, COL-I, or FN. Data represent mean values of triplicates ± SD. Similar results were obtained in three separate experiments. (TIF) [file pone.0129704.s003.tif]

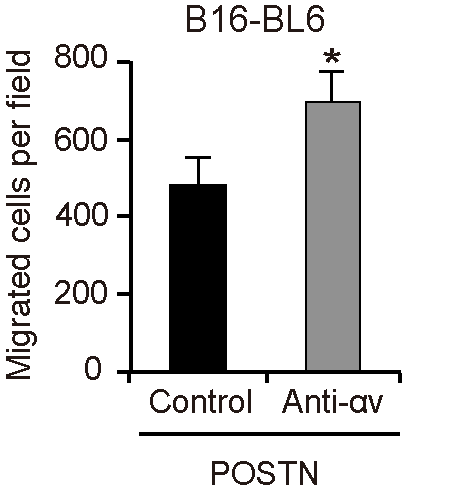

Supplement: S4 Fig — Transwell migration assay for B16-BL6 cells cultured in wells or on culture inserts coated with POSTN with anti-integrin αv Ab or control Ab. Data represent mean values of triplicates ± SD. Similar results were obtained in three separate experiments. *P < 0.05. (TIF) [file pone.0129704.s004.tif]

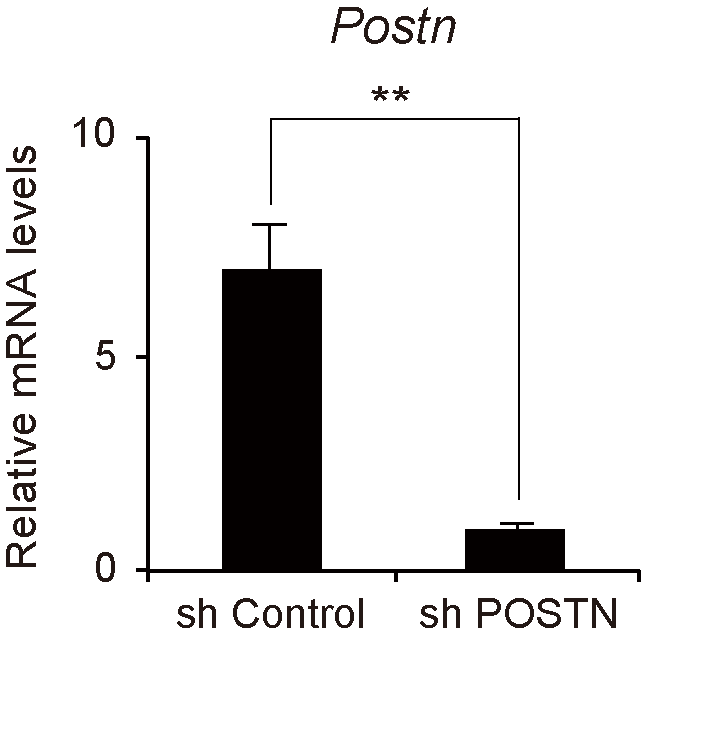

Supplement: S5 Fig — The expression of Postn mRNA in MC3T3-E1 cells stably expressing control or POSTN shRNAs was measured by quantitative RT-PCR analysis. Data are means ± SD for triplicate experiments. **P < 0.005. (TIF) [file pone.0129704.s005.tif]

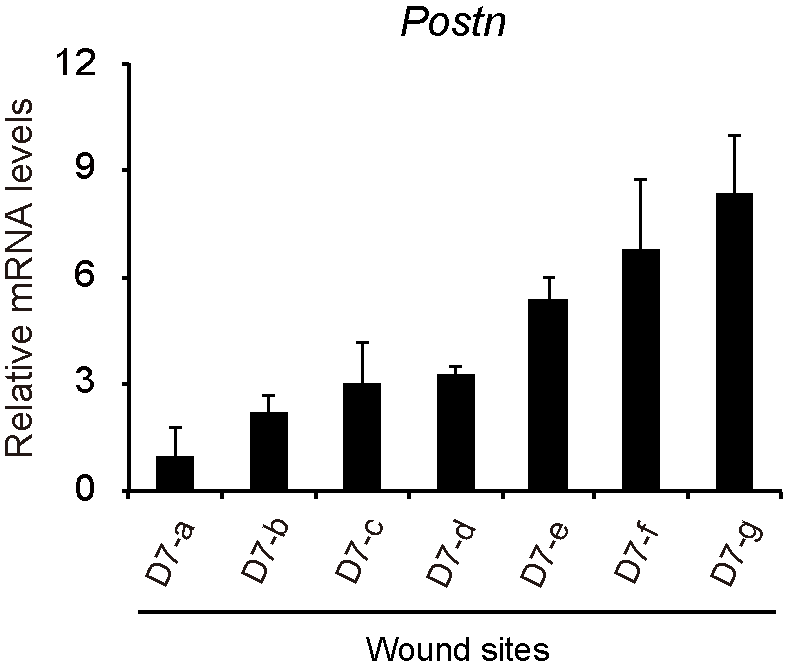

Supplement: S6 Fig — Expression of Postn mRNA at the wound site 7 days post wounding in 7 mice (designated D7-a, D7-b, D7-c, D7-d, D7-e, D7-f, and D7-g) was measured by quantitative RT-PCR analysis. Data are means ± SD from one experiment representative of three independent experiments. (TIF) [file pone.0129704.s006.tif]

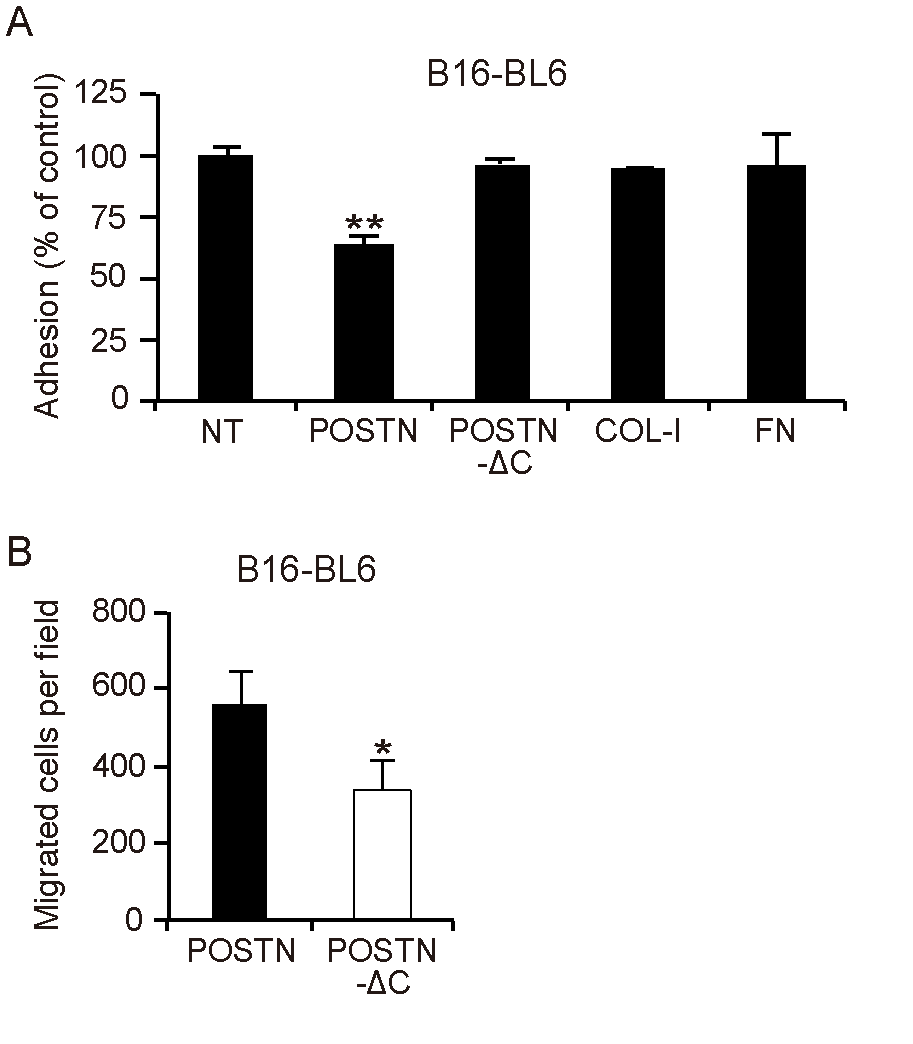

Supplement: S7 Fig — (A) Adhesion assay for B16-BL6 and cells cultured in wells or on culture inserts coated (or not, NT) with POSTN, POSTN-ΔC, COL-I, or FN. Data represent mean values of triplicates ± SD. Similar results were obtained in two separate experiments. **P < 0.005 vs. NT (B) Transwell migration assay for B16-BL6 cells cultured in wells or on culture inserts coated with POSTN or POSTN-ΔC. Similar results were obtained in two separate experiments. *P < 0.05. (TIF) [file pone.0129704.s007.tif]

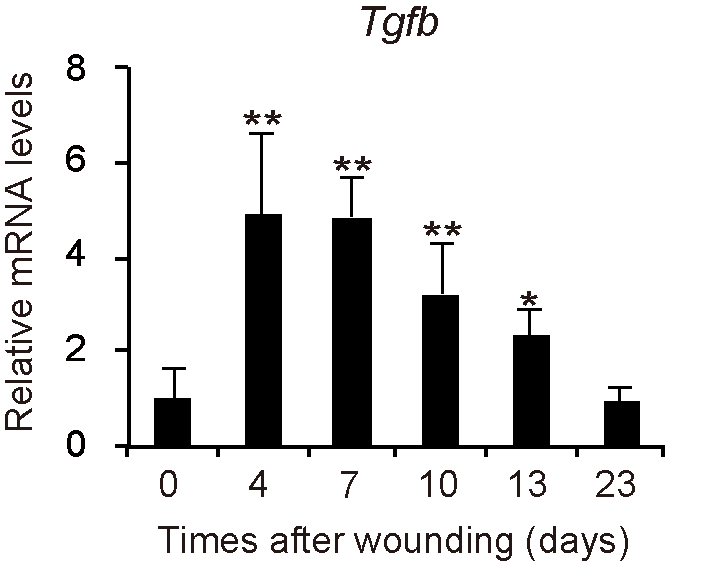

Supplement: S8 Fig — The abundance of Tgfb mRNA at the wound site 4, 7, 10, 13, or 23 days post wounding (n = 6 each) was determined by quantitative RT-PCR analysis. Intact skin (baseline, 0 days) was examined as a control. Data are means ± SD from one experiment representative of two independent experiments. *P < 0.05, **P < 0.005 vs. intact skin. (TIF) [file pone.0129704.s008.tif]
